# The use of FOCUS Harmonic scalpel compared to conventional haemostasis (knot and tie ligation) for thyroid surgery: a prospective randomized study

**DOI:** 10.1186/2193-1801-3-639

**Published:** 2014-10-28

**Authors:** Matteo Angelo Cannizzaro, Salvatore Lo Bianco, Laura Borzì, Andrea Cavallaro, Antonino Buffone

**Affiliations:** Department of “Scienze Chirurgiche, Trapianti d’Organo e tecnologie Avanzate”, University of Catania. Endocrine surgery Unit, “Policlinico-Vittorio Emanuele” Hospital, Catania, Italy

**Keywords:** Total thyroidectomy, Harmonic

## Abstract

Haemostasis is crucial in thyroid surgery to avoid intraoperative and postoperative complications. In the present study, we evaluated the efficiency and the safety of Harmonic scalpel when compared to conventional suture ligation in open total thyroidectomy. We enrolled 265 patients who underwent total thyroidectomy for multinodular disease since October 2011 up to October 2013. They were randomized into two groups: 141 in group HS (Harmonic Scalpel), 124 in group CT (Conventional tecnique). We recorded the following data: operative time, post-operative blood loss, length of hospital stay and complications. The patients were monitored for 48 hours after surgery. Several differences were observed between the two groups (HS vs CT): the use of Harmonic scalpel was associated to a significant reduction of surgical operative time (110 min in CT vs 79.36 min in HS, p = 0.00001) and also associated to a lower blood loss (97.38 ml in CT vs 68.72 ml in HS, p = 0.00001). The length of stay was significantly shorter in the HS group (2.75 days in CT vs 1.93 days in HS) Complication rate was similar in the two groups. According to our experience, the Harmonic scalpel represents a safe alternative to conventional haemostasis in thyroid surgery, allowing for a significant reduction of operative time, blood loss and hospitalization. The rate of complication demonstrated no significant difference among the two groups.

## Introduction

The technique of thyroidectomy was born in 19^Th^ century (Hannan 2006), but during the last year various technique of hemostasis, neuromonitoring and surgical excision (MiVATT, loupes assisted, endoscopic, robotic, ToVAT) are invented. Total thyroidectomy is performed in several thyroid diseases and a meticulous hemostasis is important to preserve the parathyroid glands and recurrent nerves. In the traditional technique, the hemostasis relies on knot tying and electrocoagulation. Whereas suture ligation is a time-consuming procedure, the electrocautery implies the potential risk of injuring surrounding tissues because of heat dispersion. In the 1990, the Harmonic scalpel was developed by Ethicon as an alternative to conventional hemostatic tecniques: the device allows simultaneous cutting and coagulation of vessels by using mechanical vibration at a frequency of 55.5 KHz. Although it was introduced for abdominal and laparoscopic surgery (Laycock et al. 1996; Amaral 1994), the Harmonic scalpel device demonstrated some advantages in thyroidectomy in several published studies (Bandi et al. 2008; Ferri et al. 2011; Ecker et al. 2010).

The aim of this prospective randomized study is to evaluate the real benefits of the Harmonic when compared with conventional ligation, in terms of operative time, postoperative blood loss, length of stay and complications.

## Materials and methods

We enrolled 265 patients who underwent total thyroidectomy for multinodular disease from October 2011 to October 2013. Patients (n = 10) with toxic disease, intrathoracic goiter, malignant disease who required central or lateral lymphadenectomy were excluded from the study.

**Figure 1:**
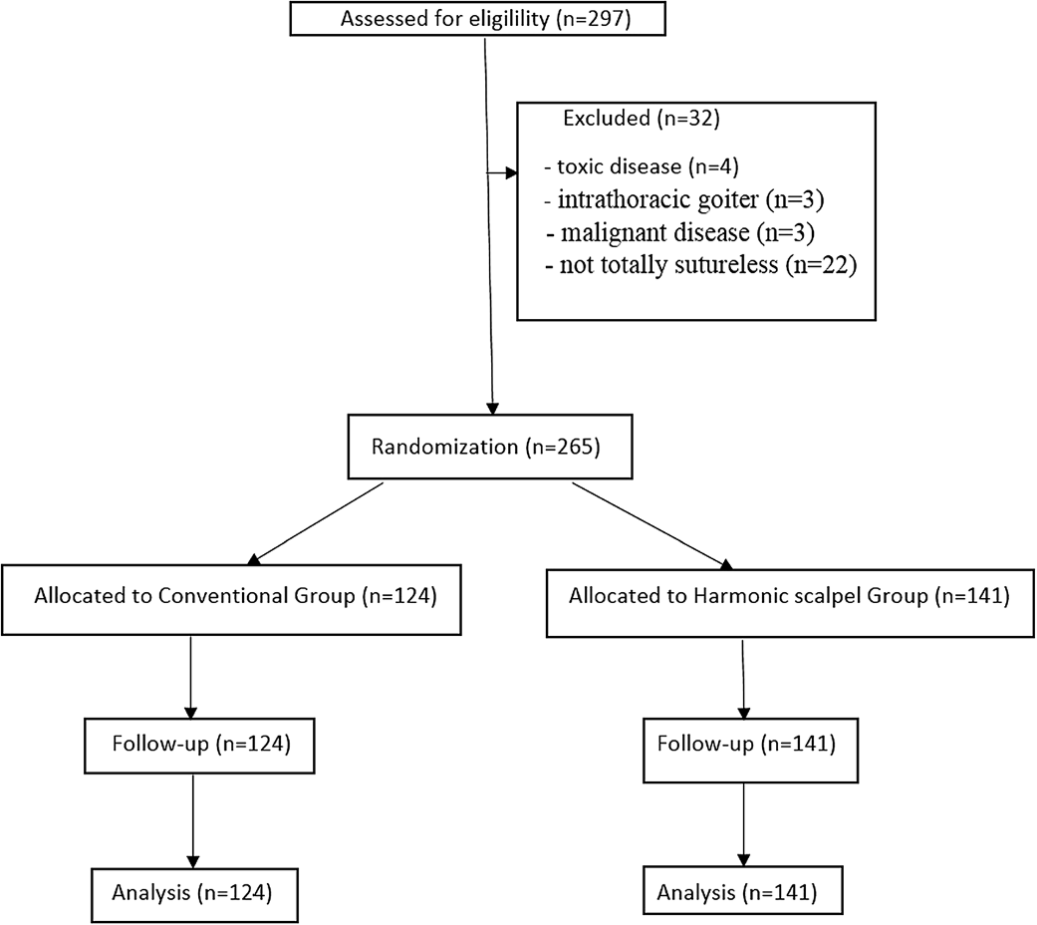
**Flow diagram.**

Twenty two cases because of topographic reasons (close proximity between inferior laryngeal nerve and vascular structures to seal) were excluded because the surgical technique adopted was not totally sutureless.Patients were randomized, with the method of sealed envelope, into two groups according to the haemostatic technique: 124 patients were included in Conventional group (CT), in whom dissection and hemostasis were performed using conventional materials (Vicryl 3-0/4-0, stitches, V titanium hemostatic clips and monopolar or bipolar electrocautery); 141 were included in Harmonic scalpel group (HS), in which the Harmonic Focus was used: the application of ultrasound to tissues was performed during the enterely procedure, to obtain three purposes synergistically: coagulation, cutting, and cavitation (Figure 1).

Mean age of patients was of 53 years (range 12-81) and the M/F ratio was of 1: 6,25. The distribution between the two groups was comparable.

All the patients underwent to otorhinoloaryngologic control and ultrasound control preoperatively.

All the surgical procedures were performed under general anesthesia by the same surgeon. The cervicotomy was performed according to Kocher technique with a length of incision of about 4-6 cm. Either preoperative and postoperative data were collected by the surgical staff.

The clinical endpoints of this study included intraoperative and post-operative parameters. Intraoperative parameters included operative time (between skin incision and the end of wound closure) and blood loss (blood loss was measured from the increase in weight of the bloodied swabs or measured from intraoperative drainage). Postoperative parameters included volume of drainage fluid in millimeters at 48 hours after surgery, drainage was maintained for 48 hours in some cases due to the presence of blood, (Abdovac^TM^ FG 14 vacuum drain circuit: average pressure -25 mmHg/3.3 kPa), serum calcium and PTH values (mg/dl) measured either preoperatively and 48 hours after surgery, hypocalcemia (temporary or permanent), wound complication (such as seroma and hematoma), RLN palsy (temporary or permanent), and length of hospital stay. Hypocalcemia was defined as permanent when it was associated with a need for calcium replacement after six months. The preoperative evaluation and the postoperative follow-up of vocal cord mobility was obtained via laryngoscopy performed prior to the surgery and 1–2 days after the surgery. Any reduction in vocal cord movement was recorded as postoperative cord paralysis. Recurrent laryngeal nerve palsy was considered permanent when it persisted more than 6 months after surgery.

Research was carried out according to the institution’s ethical guidelines and all patients gave informed consent to take part in the study. The study was approved by Department of “Scienze Chirurgiche, Trapianti d’Organo e Tecnologie Avanzate” University of Catania.

### Statistical analysis

The data collected were subjected to statistical evaluation by software StatSoft STATISTICA v.10 Continuous variables were calculated and reported as mean + SD. Categorical variables were described using frequency distributions. The Student’s t test was used to detect differences in the means of continuous variables, and chi-square test was used in cases with low expected frequencies (p < 0.05 was considered to be significant).

## Results

### Preoperative parameters

**Figure 2:**
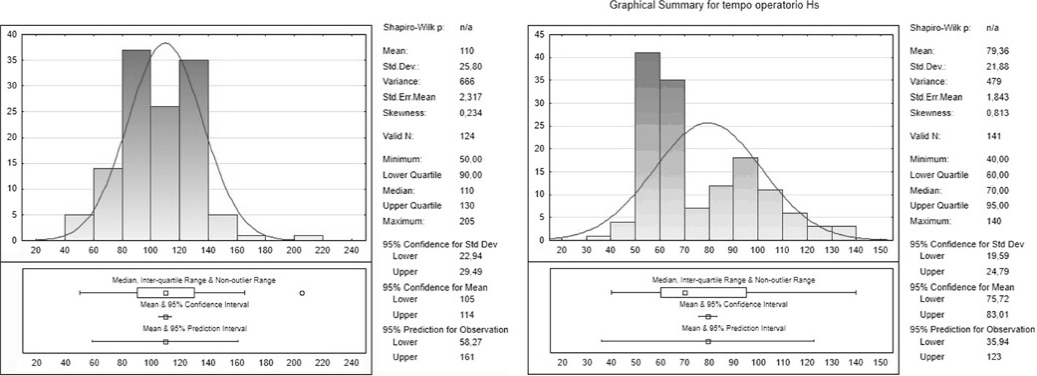
**Operative time.**

The surgical time was significantly shorter in the harmonic group (HS) than in the conventional technique group (CT): 79.36 ± 21.88 min vs 110 ± 25.80 respectively; t =6.5, P < 0.00001 (Figure 2).

### Postoperative parameters

**Figure 3:**
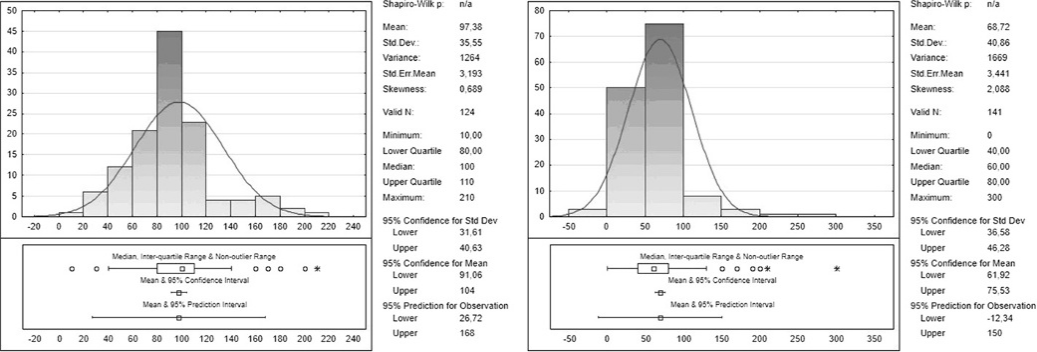
**Post-operative blood loss.**

**Figure 4:**
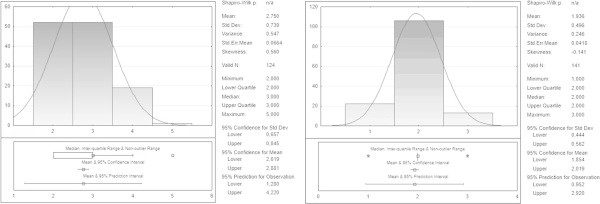
**Hospital stay.**

The post-operative blood loss volume appeared to be further reduced in HS group (Figure 3), with an average of 68.72 ± 40.86 ml vs 97.38 ± 35.55 ml in the CT group (t =5.73, p <0.00001).The length of hospital stay was shorter (Figure 4) in the HS group vs CT group (1.93 ± 0.496 days vs. 2.75 ± 0.739, t =9.44, p <0, 00001). The serum calcium levels at 12 h and 48 h showed no statistically significant differences in the two groups (respectively 7.51 ± 0.59 and 8.13 ± 0.51 group F vs 7,94 ± 0.49 and 8.23 ± 0.32 in the group T, p >0.05).

In either groups, there were no cases of permanent postoperative hypocalcemia.

Twenty seven (27) cases of temporary hypocalcemia have been reported, 14 in the HS group and 13 in the CT group.

No significant difference for temporary RLN palsy rate was found between groups (only 1 case in each group which resolved after 48 hours endotracheal extubation). We experienced no case of permanent RLN paralysis.

## Discussion

The thyroid is an high vascularized gland which receive arterial blood from the superior and inferior thyroid arteries. Therefore a careful hemostasis is crucial in order to avoid some complications as hematomas and/or seromas that sometimes may cause a potentially lethal asphyxia (Rosato et al. 2004). The use of the conventional technique requires more time to perform an adequate haemostasis.

Over the years new instruments were introduced, such as monopolar or bipolar electrocoagulation: they have demonstrated the disadvantage of thermal diffusion that could damage adjacent structures and especially the inferior laryngeal nerves.

The Harmonic Scalpel is the latest ultrasonic device designed for thyroid surgery, offering significant benefits in the size and weight of its hand piece, hand-activated trigger system, and versatility.

Shemen (2002) has largely demonstrated the effectiveness of the Harmonic scalpel in thyroidectomy analysis of 105 cases. Siperstein et al. (2002) have successfully used the Harmonic Scalpel to hemostasis of all the vessels also describing the technique of “double bind” that consists in the double coagulation of two successive areas of the vessel.

The advantages of using the Harmonic scalpel have been demonstrated also in our study, as described above. Is necessary to underline that great care should also be taken in consideration when in close proximity to delicate structures such as the inferior laryngeal nerves.

Also when performing thyroid surgery the collaboration of the entire surgical team is important: in our experience the surgeon usually highlights the vase to tie and the first assistant applies the Harmonic scalpel. The use of drainage after thyroidectomy is still a source of debate, in fact Papavramidis et al. (2014) in his scientific research has concluded that there is no advantage or disadvantage in the use of drainage after thyroid surgery. The incidence of hematoma not change with both the drainage that without drainage, and also in cases where there is a relative increase in the incidence of hematoma this is attributable to the size of the gland. It is also seen that the onset of hematoma after 24 h occurs mostly in patients who have undergone resection of a substernal goiter or with cardiac comorbidities. So in conclusion it was found that the drainage only gives discomfort to the patient and which is used for the serenity of the surgeon. According to our study we observed a difference in operative time (about 20 minutes shorter in the HS group) and a shorter length of hospital stay, as also demonstrated by Papavramidis et al. (2014), likely due to lower amount of blood loss collections when compared to the conventional technique. There were no cases of postoperative hypocalcemia in either groups (Costanzo et al. 2010), in fact, the values of serum calcium and parathyroid hormone, which are considered predictors of hypocalcemia (Cordon et al. 2005; Koutsoumanis et al. 2007; Grodski and Serpell 2008), were within standard values. This may lead to cost advantages because of the shorter length of hospital stay and shorter time of anesthesia duration/operatory room use thus increasing the number of daily procedures (Cannizzaro et al. 1993; Melck and Wiseman 2010; Duan et al. 2013).

## Conclusions

According to our results, the Harmonic scalpel reduces the operative time, postoperative blood loss and length of hospital stay. In contrast some postoperative parameters (serum calcium at 12 and 48 hours, hypocalcemia, RLN palsy) showed no statistical difference.

Therefore we conclude that the use of Harmonic scalpel during surgery for total thyroidectomy is advantageous, however a scrupulous and careful care of the surgical technique and hemostasis should always be observed.
